# Development and Evaluation of a Screening Scale for Indirect Trauma Caused by Media Exposure to Social Disasters

**DOI:** 10.3390/ijerph18020698

**Published:** 2021-01-15

**Authors:** Eun Young Choi, Seung-Hye Choi, Haeyoung Lee

**Affiliations:** 1 Department of Nursing, Graduate School of Chung-Ang University, Seoul 06974, Korea; 11351@naver.com; 2 College of Nursing, Gachon University, Incheon 21936, Korea; 3 Red Cross College of Nursing, Chung-Ang University, Seoul 06974, Korea

**Keywords:** social disaster, indirect trauma, indirect victims, PTSD, media

## Abstract

As a result of mass media development, disaster-related information, such as the severity of damage, can be easily shared; thus, the issue of consequent indirect trauma has become as important as that of direct trauma. This study developed a scale to measure the degree of indirect trauma caused by media exposure to social disasters and then verified this scale’s reliability and validity. Initial items were developed through a literature review; 39 items were selected by examining their content validity and conducting a pretest. To verify the scale’s validity and reliability, exploratory factor analyses were conducted, and Cronbach’s alpha coefficients were calculated. The explanatory power of the screening scale developed through this study was 62.2%. The scale was ultimately composed of three factors comprising 24 items. Through exploratory factor analyses, factors were identified as “psychological, physical, and behavioral responses to social disasters” (factor 1), “moral resentment due to social disasters” (factor 2), and “a sense of threat to life due to social disasters” (factor 3). Regarding internal reliability, Cronbach’s alpha values ranged between 0.85 and 0.96. Future studies with expanded participant populations are suggested, which could further verify the scale’s validity and reliability and complement its shortcomings.

## 1. Introduction

A disaster can be defined as “a serious disruption of the functioning of the community or a society causing widespread human, material, economic or environmental losses which exceed the ability of the affected community or society to cope using its own resources” [1]. Disasters are classified into natural disasters, such as typhoons, floods, and earthquakes, and social disasters, including fires, building collapses, explosions, traffic accidents (including aviation and marine accidents), and chemical, biological, or nuclear accidents [2]. Disasters can threaten lives, cause economic losses, and damage both mental and physical health [3]. The psychiatric disorder most frequently experienced by disaster survivors is post-traumatic stress disorder (PTSD). PTSD has become an important issue because of the large number of traumatic events that have been occurring, such as terrorist attacks or meteorological disasters. PTSD is characterized by four main dimensions: re-experience, persistent avoidance, arousal after a traumatic event, and cognitive and mood disturbances [4]. Moreover, survivors may also experience acute stress disorder, major depressive disorder, and anxiety disorders [5]. Whereas disaster survivors’ physical injuries can be identified and treated immediately, the treatment of mental health problems that are not outwardly apparent is likely to be delayed or overlooked [6].

Regarding mental health problems that can occur after trauma, the characteristics of the traumatic events are important, as are individual subjective perceptions of the events [7]; thus, the risk factors for post-traumatic stress may be present not only following direct trauma, but also for indirect trauma. In modern society in particular, various media sources allow disaster-related information, such as the severity and degree of damage, to be shared easily, even among those who are not directly impacted by the disaster. Therefore, the frequency of indirect exposure and the amount of available information have increased, thereby increasing the possibility that indirect trauma will lead to stress, anxiety, or depression [8].

Indeed, many previous domestic and foreign studies reported that exposure to disasters through mass media is related to vicarious trauma experiences and serious psychological trauma [9,10,11,12,13]. In particular, findings that there were no differences between levels of PTSD in those who experienced a tragic disaster firsthand, such as the 9/11 terrorist attacks, and those who were indirectly exposed to the disaster through television-provided supporting evidence for the importance of media-mediated effects from disasters [14]. Furthermore, previous research presented important implications regarding indirect trauma. For example, one study reported that repeated exposure to television broadcasts that vividly convey disaster scenes has larger negative consequences than direct exposure to traumatic events [15], while another study reported that social media channels also lead to PTSD symptoms similar to those engendered by television broadcasts [16]. Additionally, Silver et al. [12] reported that exposure via television to the initial 9/11 terrorist attacks and subsequent Iraq war and exposure frequency were predictors of PTSD symptoms 2–3 years later, and exposure to 9/11-related television broadcasts for more than 4 h a day and accumulated acute stress were predictive of physical illness 2–3 years later.

Disasters can be neither easily predicted nor avoided but can happen anytime, anywhere, to anyone. Therefore, they are problems that humanity must overcome, and the paradigm for coping with disasters has recently shifted from vulnerability to resilience [17]. Post-traumatic stress has lasting effects on individuals and society, leading to large increases in direct and indirect costs and providing a major obstacle to community recovery following a disaster [18]. Therefore, identifying disaster survivors at high risk for PTSD and classifying them appropriately to prioritize their treatment is the first step in protecting and systematically managing the mental health of disaster survivors [6]. Although this is important for those exposed both directly and indirectly to disasters, since the scale of indirect victims cannot be specified, a scale that will enable screening for individual degrees of indirect trauma should be developed to identify high-risk groups.

To date, various tools that can assess individual mental health following a disaster have been developed [19], including scales for measuring PTSD, depression, and anxiety. Additionally, tools for measuring suicidal ideation or sleep disorders have been generated, and there are also scales to evaluate other symptoms (e.g., anger response, anger rumination) and complex disorders. However, when screening for the degree of indirect damage caused by trauma, it may be difficult to apply existing tools or to properly filter the concepts to be measured, if there are too many scales or they are too complex. Moreover, when measuring trauma caused by social disasters, not only sociocultural differences but also experiences before and after a disaster should be considered, such as the method and amount of media exposure [19]. Furthermore, although it has been reported that indirect trauma has a similar structure and symptomology to PTSD [20], a scale that reflects the unique characteristics of social disasters, such as moral resentment [21], is necessary to properly measure indirect trauma. Therefore, this study developed and verified the validity and reliability of the Scale for Indirect Trauma caused by Media Exposure to Social disaster (SITMES).

## 2. Materials and Methods

**Figure 1:**
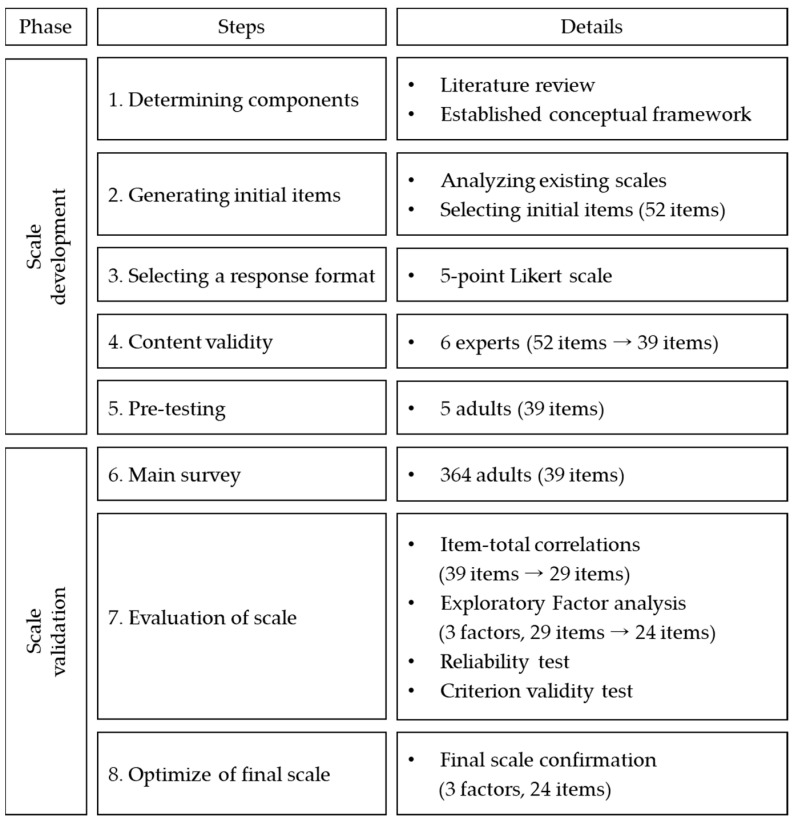
Steps of scale development and validation.

This study was conducted in two phases: scale development and verification (Figure 1).

### 2.1. Scale Development

#### 2.1.1. Determining Components

To identify scale components, previous studies related to social disasters were reviewed [13,16,22,23,24,25,26,27,28,29,30]. According to the literature review, the symptoms of indirect trauma caused by media exposure to social disasters were identified as psychological, physical, and behavioral responses. However, it was found that even when people experienced the same disaster, factors such as individual characteristics and social support affected the manifestation of trauma symptoms. In particular, in cases where disasters were indirectly experienced through the media, degrees of trauma symptoms differed according to the type and amount of media exposure. However, as existing trauma assessment tools mainly measure symptom severity, efforts were made in the present study to include factors that affect trauma symptoms along with trauma symptoms.

In this study, the conceptual framework was constructed using four concepts based on the Disaster Reintegration Model [22], which emphasizes the importance of resilience in individuals and public support systems to recover from the effects of disasters. Additionally, the 11 components were determined by referring to variables shown to be effective in Lee et al. [26], who provided a meta-analysis of disaster-related variables based on an ecological model.

The four concepts and 11 components were as follows: (a) exposure and response to events (media exposure, psychological symptoms, physical symptoms, and perceived sense of threat to life), (b) individual resilience (trauma experience before the event, stress coping methods, negative emotional responses, and meaning in life and problem-solving), (c) support system (family support and social support), and (d) public resilience (use of information and services).

#### 2.1.2. Generating Initial Items

To develop the initial items, 21 measures related to each component were analyzed [11,13,16,23,24,30,31,32,33,34,35,36,37,38,39,40,41,42,43,44,45] (Supplementary Materials). In analyzing each item, the appropriate items were selected for each component. Among them, statements with similar meaning were integrated and the wording modified to suit the purpose of this study. Items related to “use of information and services” were described by the researchers based on Choi et al. [22]. A total of 52 initial items were generated, which were divided among components as follows: media exposure (3), psychological symptoms (7), physical symptoms (3), perceived sense of threat to life (4), trauma experience before the event (3), stress coping methods (10), negative emotional responses (5), meaning in life and problem-solving (4), family support (4), social support (10), and use of information and services (3).

#### 2.1.3. Selecting a Response Format

The initial items were measured using a 5-point Likert scale (1 = “Strongly disagree”, 2 = “Disagree”, 3 = “Neutral”, 4 = “Agree”, 5 = “Strongly agree”), with higher scores indicating higher risk of indirect trauma from media-based exposure to social disasters.

#### 2.1.4. Content Validity

Content validity of the initial items was reviewed by six experts, consisting of three professors who specialized in mental health nursing, one professor with extensive experience in scale development, and two clinical counseling experts. The expert group was asked to evaluate how appropriately each item measured the degree of indirect trauma caused by media exposure to social disasters using a 4-point Likert scale (1 = “Not at all”, 2 = “Not much”, 3 = “A little”, and 4 = “Very much”), and to review each item’s readability and appropriateness of expression.

As a result of the experts’ review, eight items with a content validity index (CVI) lower than 0.80 were deleted, and five items with similar meanings were integrated with other items; thus, 39 items were selected in total. The excluded items mainly related to media exposure, trauma experiences before the event, and stress coping methods. Regarding components, media exposure and trauma experiences before the event were integrated into psychological symptoms, meaning in life was integrated into negative emotional responses, and the use of information and services was integrated into problem-solving. The number of items for the modified eight components was as follows: psychological symptoms (9), physical symptoms (3), perceived sense of threat to life (3), negative emotional responses (5), dysfunctional coping methods (5), problem-solving (5), family support (4) and social support (5).

#### 2.1.5. Pretesting

A pretest was conducted to identify how well the scale’s items could be understood and to evaluate their applicability. Five participants were selected for each age group from the 20s to the 60s, using convenience sampling. The participants provided descriptive feedback regarding items that were difficult to understand or should be corrected.

### 2.2. Scale Validation

#### 2.2.1. Participants and Procedure

The study participants included in the validity and reliability analysis of the developed preliminary scale were adults from the general population (age range 20–64 years) who were exposed to social disasters through various forms of media. The exclusion criteria were as follows: (1) people directly exposed to social disasters; (2) people who had not been directly exposed to social disasters, but had a family member, friend, or acquaintance who suffered direct damage from such an event; (3) workers in occupations related to rescue or recovery during social disasters (e.g., medical workers, nurses, mental health professionals, emergency crews, firefighters, police officers), and (4) people who lived in areas where social disasters occurred and who provided assistance to or experienced grief with survivors. To filter out participants who met the exclusion criteria, the screening questions were answered at the beginning of the online survey. The recommended sample size for factor analysis is 10 respondents per survey item and/or 200–300 observations [46,47,48,49]. In particular, several studies have suggested that a sample size of over 300 is desirable [50,51]. Therefore, we aimed to sample more than 330 respondents, given potential dropout.

Data collection was performed by an approved institution that had no research interest and complied with the code of ethics and research methods of the Korean Association for Survey Research and the Statistic Acts. The survey was conducted from February 19 to 28 February 2020, using an online questionnaire, and the participants were recruited using a survey panel. A total of 377 respondents took the survey, and 364 were included in the final analysis, after 13 questionnaires were excluded due to participants responding based on events other than social disasters (e.g., natural disasters, murders, criminal incidents).

#### 2.2.2. Measurements

To verify criterion-related validity of preliminary scales, we used the Korean version of the Impact of Event Scale-Revised (IES-R-K) [52], and the Korean version of the Connor–Davidson Resilience Scale (K-CD-RISC) [53]. The IES-R-K has a total of 22 items, each of which is evaluated on a scale ranging from 0 to 4 points; total scores are calculated by summing individual item scores and range from 0 to 88 points. The internal reliability of the scale was Cronbach’s α = 0.93 originally and 0.98 in this study. The K-CD-RISC has a total of 25 items, and each is evaluated using a 5-point scale (0–4 points) [46]. Total scores range from 0 to 100 points and are calculated by summing individual item scores; the mean value was analyzed. The internal reliability of the scale was Cronbach’s α = 0.93 originally and 0.94 in this study.

Demographic characteristics included gender, age, level of education, average monthly income, cohabitation status, and employment status. Information regarding exposure to social disasters included the most recent social disaster reported through the media, time elapsed since exposure to the incident, amount of media usage to obtain information regarding the incident, and other psychological difficulties present before exposure to the incident.

#### 2.2.3. Statistical Analysis

The data were analyzed using SPSS/WIN 25.0 (IBM Corp., Armonk, NY, USA). Item-level analysis consisted of inter-item and item-total correlations. Items with very low correlations (<0.30) were dropped from the tentative scale [54]. To test construct validity, we used exploratory factor analysis (EFA). The Kaiser–Meyer–Olkin measure of sampling adequacy (KMO) and Bartlett’s test of sphericity were used to examine the suitability of the data for EFA. The KMO is an index used to compare the values of observed correlation coefficients with values of partial correlation coefficients; the larger the value, the more common potential factors underlie the measurement variables [55]. In Bartlett’s sphericity test, the hypothesis (H0: The correlation coefficient matrix is a unit matrix) was two-sided, tested at a statistical significance level of 0.05. EFA used a maximum likelihood (ML) factor extraction method with direct oblimin rotation to find factor patterns that could be interpreted as factors. We also considered Kaiser’s eigenvalue rule [56], scree plots [57], and the minimum average partial (MAP) test [58] to determine the number of factors. Subsequently, items with factor loadings below 0.4 or items with cross loadings greater than 0.3 were dropped [54]. To verify the reliability of the scales, Cronbach’s alpha values were obtained. Pearson’s correlations were obtained between the factors of SITMES, IES-R-K, and K-CD-RISC to verify the criterion validity of SITMES. Correlations between individual scores were analyzed, and scales with higher degrees of correlation (larger correlation coefficients) were judged to have higher criterion validity. The data on participants’ demographic characteristics and information on exposure to social disasters were analyzed using descriptive statistics, and the differences in SITMES scores according to participants’ demographic characteristics and information on exposure to social disasters were analyzed using t-tests and one-way ANOVAs. Post hoc analyses were conducted using Scheffé’s test.

### 2.3. Ethics

The study was approved by the Institutional Review Board (IRB no 1041078-201912-HRSB-376-01C) of the C university. Potential ethical issues were addressed, including plagiarism, informed consent, misconduct, data fabrication and/or falsification, double publication and/or submission, and redundancy.

## 3. Results

### 3.1. Participants’ Characteristics

Of the 364 total participants, 188 (51.65%) were men and 176 (48.35%) were women; the mean participant’s age was 40.49 ± 10.99 years. Regarding level of education, no participant had a middle school graduation or lower level, and college graduates accounted for the largest percentage (65.11%). In terms of average monthly income, the largest group included those with an income of 3–6 million won (42.48%). Most participants lived with family members (84.62%), while 13.19% lived alone. Regarding employment status, wage earners were the group with the highest percentage (63.74%).

Among the most recent social disasters reported in the media, infectious diseases/viruses accounted for the highest percentage (48.35%), followed by accidents such as airplane collisions/ship accidents (37.64%), fires/building collapses (8.79%), nuclear accidents/industrial disasters/toxic substances accidents/hydrogen explosions/gas explosions (3.57%), and violent situations (1.65%). The largest group of participants recollected an event that occurred within the previous month (61.54%), followed by 1–3 months (25.55%). Regarding the duration of time over which media sources were used to obtain information related to the incidents, the response “a little longer (compared to normal)” was the most frequent (33.79%). Regarding whether respondents experienced psychological difficulties related to other incidents before the relevant incident, 24.45% and 75.55% answered “yes” and “no”, respectively (Table 1).

### 3.2. Evaluation of Scale

#### 3.2.1. Item Analysis

The correlation coefficients between all items and an individual item were obtained. Items with a correlation coefficient of 0.3 or less and items with inverse correlations were deleted, as they had a weak contribution. As a result, 10 items (26, 27, 28, 29, 30, 31, 32, 34, 36, 37) with inverse correlations were deleted. Inter-item correlations for 29 items ranged from 0.22 to 0.81.

#### 3.2.2. Construct Validity Test

First, EFA was conducted with 29 items. The factor analysis produced three factors with eigenvalues >1 (15.759, 2.395, 1.054) and the cumulative variance explained was 62.2%. The scree plot showed that the slope of the curve exhibited a discontinuity at three factors and the MAP test result also supported the existence of three factors. Thus, we selected three factors.

In the first EFA, the KMO value was 0.97 and the Bartlett’s test statistic value was 8882.47 (*p* < 0.001). In the pattern matrix, there were no items with factor loadings below 0.4, but items 4, 6, and 17 were deleted, as they exhibited factor loadings of 0.3 or higher on more than two factors.

The second EFA was conducted with 26 items. The KMO value was 0.96 and Bartlett’s test statistic was 7518.42 (*p* < 0.001). In pattern matrix, items 15 and 25 were deleted, due to factor loadings below 0.4, but no items exhibited cross factor loadings of 0.3 or higher.

The third EFA was conducted with 24 items. The KMO value was 0.96 and Bartlett’s test statistic was 7042.93 (*p* < 0.001). In this round, all items met the factor loading criteria. The results of the final EFA are shown in Table 2.

#### 3.2.3. Factor Naming

Items were analyzed by factor to name the factors, with 15 items grouped into factor 1, which explained the largest proportion of variance. The items of factor 1 were those used in previous studies to measure physical symptoms, psychological symptoms, dysfunctional coping methods, and the effects of media exposure or traumatic experiences. Therefore, factor 1 was named “psychological, physical, and behavioral responses to social disasters.” Factor 2 comprised five items, including those used to measure negative emotional reactions and anger and skepticism toward society, among those measuring dysfunctional coping methods. Therefore, factor 2 was named “moral resentment due to social disasters.” Factor 3 comprised four items, including those used to measure psychological symptoms and perceived threat to life. Therefore, factor 3 was named “a sense of threat to life due to social disasters.”

#### 3.2.4. Reliability Test

Regarding internal consistency reliability of the 24 items of SITMES, Cronbach’s α = 0.96. Inter-item reliability for the scale’s three factors was analyzed; the results indicated high reliability: Cronbach’s α = 0.96, 0.89, and 0.85 for factors 1, 2, and 3, respectively (Table 3).

#### 3.2.5. Criterion Validity Test

The correlations between indirect trauma and the three factors measured with the finalized version of SITMES, IES-R-K, and K-CD-RISC are shown in Table 4. Scores of SITMES showed a significant correlation with the results of the IES-R-K (*r* = 0.69, *p* < 0.01); however, SITMES scores had no significant correlation with K-CD-RISC.

### 3.3. Differences in SITMES Scores According to Participant’s Characteristics

The differences in SITMES scores according to participants’ demographic characteristics and information on exposure to social disasters were analyzed. Accordingly, no differences were found in SITMES scores according to gender, level of education, or income level; however, there were significant differences according to age (F = 3.62, *p* = 0.01), as older age was positively associated with greater SITMES scores. When differences in SITMES scores according to incident type were analyzed, the results indicated that the SITMES scores related to an “infectious disease/virus” were greater than those for “airplane collision/ship accidents/traffic accidents” (F = 5.35, *p* ≤ 0.01). SITMES scores were also higher among participants who spent more time using media to obtain information related to an incident, compared with those who spent less time on this activity (F = 20.03, *p* ≤ 0.01). SITMES scores were higher for those who had experienced psychological difficulties before an incident than among those who had not (F = 24.68, *p* ≤ 0.01; Table 1).

## 4. Discussion

The SITMES, developed in this study, consists of three factors and 24 items. The first factor was “psychological, physical, and behavioral responses to social disasters”, comprising 16 items, which was identified as the most important factor because it explained the largest proportion of response variance. This was thought to be attributable to trauma symptoms appearing in mixture in diverse aspects. According to a previous study, media-mediated experiences of disasters emerge via physical and physiological stress activation mechanisms. That is, when people are shown images of traumatic events by media, fear circuits of the brain activate and generate flashbacks, which are key processes in PTSD [16]. In particular, the integration of trauma coping capabilities into this factor supports the study’s findings indicating that this factor affects the expression of trauma symptoms [12,24,26].

The second factor, consisting of five items, was “moral resentment due to social disasters.” A characteristic of emotional responses to social disasters is to experience moral resentment at the occurrence of the incident, in addition to mental health outcomes, such as PTSD. Moral resentment is an emotion that enables moral judgment and leads individuals to seek the legitimacy of punishment for unjust acts. Therefore, moral resentment causes social changes powered by righteous indignation and motivated by the desire to restore justice [21]. In particular, negative emotional responses can be expressed not only as anger but also as grudges or resentment, which can be viewed as a response similar to post-traumatic embitterment disorder [59].

The third factor was “a sense of threat to life due to social disasters”, and comprised four items. This factor was related to the belief that an incident similar to the social disaster may occur to oneself, family members, or friends, and can be seen as a result of empathizing with the victim or the victim’s family [23]. With the development of information and communications technology (ICT), modern people are connected to various media 24 h per day [16]. In an uncertain and threatening disaster crisis, people actively seek information and depend on the media to determine the cause and effects of the disaster [16]. This is consistent with a previous study indicating that media exposure can cause indirect trauma symptoms even when the relevant person is geographically and temporally far away from a social disaster [60].

The correlation between the IES-R-K and K-CD-RISC was analyzed to verify the scale’s criterion validity. The scores obtained using the SITMES showed a positive correlation with the incident impact scale scores, particularly a high positive correlation with the first factor. This indicated that the developed scale could meaningfully reflect degrees of indirect trauma caused by media exposure to social disasters. However, the developed scale did not show any correlation with resilience scores. This may be related to the items that were deleted when verifying the scale’s construct validity. The 10 deleted items with inverse correlations corresponded to problem-solving or family or social support among trauma-coping competency, and all were inverse conversion items, for which higher scores indicated higher levels of capabilities or support. These items were expected to reflect factors that reduce indirect trauma; however, they were excluded due to incongruity with other items because of their inverse conversion responses or given that the overall scores of participants were not high, the demand for problem-solving capabilities or support systems may have been low because the trauma symptoms were not serious. Moreover, this may be due to participants’ family characteristics, which were not closely examined in the present study. Family is a fundamental resource that provides comfort and support for disaster-related trauma, but in some cases, negative interactions may occur among family members [26]. The items related to family or social support were included in factor 1 (items 33, 35, 38, and 39). This is consistent with a conceptual framework in which support systems and the expression of symptoms are related. Including support factors would provide further distinction from existing tools, but since support systems were not categorized as a factor, items may need to be modified or supplemented to reveal such a factor.

As with previous studies, the degree of trauma increased as the amount of media exposure increased [19,61]. In addition, the degree of indirect trauma was greater for respondents who experienced trauma before the reported incident, compared to those without previous trauma experience. These items were deleted during expert verification of content validity because they were too ambiguous to be included in the questionnaire. However, these items should be considered when measuring the indirect trauma of social disasters, and studies reflecting them will be necessary in the future.

The results of the present study indicated that the degree of trauma in the middle-aged group (50s) was generally higher than in the younger age group (20s). This is consistent with a previous study that indicated older adults are more vulnerable to PTSD symptoms than younger adults, because the former are relatively more likely to have experienced war or personal loss [62]. However, no gender differences were found in trauma scores. Previous research showed women to be more psychologically vulnerable than men regarding degrees of trauma [19,63,64], but other studies found degrees of trauma do not differ according to gender [14,65]. This suggests that the scale developed in the present study can discriminate between groups at high and low risk for indirect trauma from social disasters; however, since the results were also shown to conflict with some previous research, such as that regarding gender differences, follow-up studies should be conducted with expanded participant groups. As the limitations of time and space are gradually disappearing due to the development of ICT, indirect trauma caused by social disasters is increasing. The disaster stressor may be a risk factor that amplifies the deleterious impact of media use on mental health [66]. The degree of indirect trauma due to media coverage of disasters is affected by various factors [16]; as such, victims may be unaware of such trauma. SITMES may contribute to determining the extent of indirect damage caused by the media and reducing such damage by enabling early detection of mental problems at the personal level and consequently promoting reduced media exposure at the societal level.

This study has the following limitations. First, given that many respondents chose “infectious disease/virus” as the social disaster they experienced, and the data were collected during a period when COVID-19 was spreading, the characteristics of indirect trauma measured in this study may have been biased toward incidents related to viral infection. Second, although participants were asked to remember a social disaster they recently experienced, the amount of time that had elapsed since the incidents differed among participants; thus, the findings may be affected by recall bias. Third, it is difficult to exclude the possibility of selection bias. A survey panel was used to recruit participants so they would not be limited to a certain group; however, studies conducted with larger participant samples are needed. Fourth, the proportion of individuals with low education in the sample was small. Individuals with low education may be relatively prone to media-induced distress. Additionally, previous real-life exposure was not controlled, nor were other stressors that may have increased the level of distress. Lastly, no cutoff point was presented for the developed scale. To use the SITMES more successfully, follow-up studies that present a cutoff point should be conducted. To overcome these limitations, it is necessary to further refine the scale through confirmatory factor analysis, confirm test-retest validity, and target more diverse social disasters. In addition, it is necessary to use longitudinal studies to verify the practical effectiveness of SITMES as a tool to measure indirect trauma due to social disasters.

## 5. Conclusions

In this study, a scale to screen for indirect trauma caused by media exposure to social disasters was developed and evaluated for validity and reliability. The SITMES, consisting of three factors comprising 24 items, was verified to be highly reliable and have appropriate construct validity. Efforts were made to include characteristics of cognitive and emotional responses to social disasters; however, since there may have been selection bias, and the characteristics and effects of social disasters as perceived by participants may have varied according to the time of the surveys, further refinement of the scale is necessary by applying it to a larger participant population and repeatedly verifying its reliability and validity. This study can hopefully contribute to improving community resilience in overcoming disasters by measuring the impact of indirect trauma experienced through media exposure to social disasters and detecting individuals indirectly affected by disasters who are likely to be otherwise overlooked.

## Figures and Tables

**Table 1:** Differences of SITMES scores according to participant’s characteristics.

Variables	Categories	*N* (%)	Total			Factor 1			Factor 2			Factor 3		
			M ± SD	F(*p*)	Scheffé	M ± SD	F(*p*)	Scheffé	M ± SD	F(*p*)	Scheffé	M ± SD	F(*p*)	Scheffé
Demographic Characteristics														
Age (years)	(1) 19–29	82 (22.53)	2.45 ± 0.84	3.62 (0.01)	4 > 1	1.93 ± 0.80	4.32 (0.01)	3, 4 > 1	2.55 ± 1.01	1.68 (0.17)		2.88 ± 1.07	3.73 (0.01)	4 > 1
	(2) 30–39	83 (22.8)	2.71 ± 0.85			2.11 ± 0.93			2.85 ± 1.05			3.19 ± 0.99		
	(3) 40–49	98 (26.92)	2.75 ± 0.88			2.29 ± 0.93			2.79 ± 1.02			3.16 ± 0.98		
	(4) 50–59	101 (27.75)	2.84 ± 0.71			2.34 ± 0.76			2.84 ± 0.93			3.35 ± 0.76		
Gender	(1) Male	188 (51.65)	2.67 ± 0.87	0.59 (0.45)		2.21 ± 0.93	0.33 (0.57)		2.72 ± 1.02	0.57 (0.45)		3.07 ± 0.96	2.96 (0.09)	
	(2) Female	176 (48.35)	2.73 ± 0.79			2.15 ± 0.81			2.80 ± 0.99			3.24 ± 0.96		
Education	(1) Elementary or less	0 (0.00)	0.00 ± 0.00	1.00 (0.40)		0.00 ± 0.00	1.03 (0.38)		0.00 ± 0.00	1.19 (0.31)		0.00 ± 0.00	1.10 (0.35)	
	(2) Middle school	0 (0.00)	0.00 ± 0.00			0.00 ± 0.00			0.00 ± 0.00			0.00 ± 0.00		
	(3) High school	47 (12.91)	2.61 ± 0.95			2.25 ± 1.00			2.58 ± 1.04			3.00 ± 1.03		
	(4) Attending college or university	41 (11.26)	2.53 ± 0.87			2.01 ± 0.84			2.59 ± 0.98			2.99 ± 1.13		
	(5) College graduation	237 (65.11)	2.75 ± 0.81			2.22 ± 0.84			2.81 ± 1.00			3.21 ± 0.92		
	(6) Graduate school	39 (10.71)	2.70 ± 0.78			2.06 ± 0.91			2.85 ± 1.02			3.18 ± 0.89		
Monthly income (KRW)	(1) Less than 1 million	43 (11.81)	2.42 ± 0.99	1.83 (0.12)		1.89 ± 0.97	2.57 (0.04)		2.64 ± 1.17	0.36 (0.84)		2.74 ± 1.18	2.61 (0.04)	
	(2) 1 million–less than 3 million	130 (35.71)	2.71 ± 0.79			2.14 ± 0.80			2.79 ± 1.00			3.22 ± 0.93		
	(3) 3 million–less than 6 million	155 (42.58)	2.75 ± 0.82			2.27 ± 0.89			2.77 ± 0.97			3.19 ± 0.92		
	(4) 6 million–less than 8 million	22 (6.05)	2.91 ± 0.77			2.48 ± 0.85			2.88 ± 0.90			3.39 ± 0.79		
	(5) More than 8 million	14 (3.85)	2.54 ± 0.82			2.00 ± 0.78			2.59 ± 1.07			3.04 ± 0.99		
Cohabitant	(1) Living alone	48 (13.19)	2.56 ± 0.90	1.30 (0.27)		2.07 ± 0.93	1.03 (0.36)		2.68 ± 1.07	0.17 (0.84)		2.93 ± 1.07	2.79 (0.06)	
	(2) Living with family	308 (84.62)	2.73 ± 0.82			2.21 ± 0.86			2.77 ± 1.00			3.20 ± 0.94		
	(3) Living with friend(s)	8 (2.2)	2.42 ± 0.71			1.88 ± 0.97			2.73 ± 1.10			2.66 ± 0.78		
Occupation	(1) Self-employed	34 (9.34)	2.73 ± 0.71	1.22 (0.30)		2.24 ± 0.77	1.30 (0.27)		2.75 ± 0.79	0.39 (0.82)		3.21 ± 0.91	1.56 (0.19)	
	(2) Wage worker	232 (63.74)	2.72 ± 0.82			2.20 ± 0.86			2.77 ± 1.00			3.18 ± 0.92		
	(3) Student	37 (10.16)	2.44 ± 0.85			1.89 ± 0.83			2.59 ± 1.01			2.84 ± 1.14		
	(4) Housewife	38 (10.44)	2.84 ± 0.88			2.30 ± 0.93			2.88 ± 1.14			3.34 ± 0.97		
	(5) Unemployed	23 (6.32)	2.66 ± 1.00			2.19 ± 1.01			2.78 ± 1.16			3.02 ± 1.04		
Information of exposure to social disaster														
The most recent incident as Social disaster through the media	(1) Infectious disease/viral infection	176 (48.35)	2.89 ± 0.76	5.35 (<0.01)	1 > 2	2.34 ± 0.85	4.21 (<0.01)	1 > 2	2.97 ± 0.93	4.36 (<0.01)	1 > 2	3.36 ± 0.87	4.45 (<0.01)	1 > 2
	(2) Traffic accidents/aviation accidents/marine accidents	137 (37.64)	2.50 ± 0.86			1.98 ± 0.82			2.55 ± 1.07			2.98 ± 1.01		
	(3) Nuclear accident/industrial disaster/toxic substance accident/hydrogen explosion/gas explosion	13 (3.57)	2.83 ± 0.95			2.38 ± 1.14			2.89 ± 0.93			3.21 ± 0.97		
	(4) Terror/bombing/violence	6 (1.65)	2.62 ± 0.74			2.49 ± 0.81			2.70 ± 0.76			2.67 ± 0.86		
	(5) Fire/building collapse	32 (8.79)	2.45 ± 0.84			2.02 ± 0.88			2.46 ± 0.95			2.85 ± 1.03		
Elapsed period since exposure of the incident	(1) Less than 1 month	224 (61.54)	2.63 ± 0.81	1.15 (0.34)		2.17 ± 0.84	0.34 (0.89)		2.63 ± 0.97	3.31 (0.01)	6 > 1	3.10 ± 0.94	0.97 (0.44)	
	(2) 1 month–less than 3 months	93 (25.55)	2.79 ± 0.83			2.16 ± 0.89			2.92 ± 1.00			3.31 ± 0.96		
	(3) 3 months–less than 6 months	7 (1.92)	2.78 ± 0.58			2.46 ± 0.69			2.57 ± 0.69			3.32 ± 0.66		
	(4) 6 months–less than 3 years	13 (3.57)	2.58 ± 0.77			2.06 ± 0.81			2.80 ± 0.70			2.87 ± 1.11		
	(5) 3 years–less than 5 years	7 (1.92)	2.78 ± 1.29			2.11 ± 1.32			3.20 ± 1.59			3.04 ± 1.22		
	(6) More than 5 years	20 (5.49)	3.00 ± 0.96			2.34 ± 1.03			3.41 ± 1.15			3.26 ± 1.07		
Amount of media usage to obtain information the incident	(1) Significantly shorter (than usual)	38 (10.44)	2.04 ± 0.82	20.03 (<0.01)	5 > 1–4 4 > 1, 3 3 > 1 2 > 1	1.60 ± 0.78	10.26 (<0.01)	5 > 1–3 4, 3 > 1	2.03 ± 0.91	18.01 (<0.01)	5 > 1–4 4 > 1	2.49 ± 1.12	19.29 (<0.01)	5 > 1–4 4 > 1, 3 2 > 1
	(2) Slightly shorter (than usual)	47 (12.91)	2.57 ± 0.88			1.95 ± 0.91			2.63 ± 1.12			3.12 ± 1.00		
	(3) Similar (as usual)	107 (29.40)	2.52 ± 0.74			2.12 ± 0.79			2.54 ± 0.85			2.90 ± 0.88		
	(4) Slightly longer (than usual)	123 (33.79)	2.84 ± 0.69			2.33 ± 0.76			2.91 ± 0.88			3.28 ± 0.81		
	(5) Significantly longer (than usual)	49 (13.46)	3.38 ± 0.77			2.63 ± 1.02			3.57 ± 0.97			3.93 ± 0.72		
Other psychological difficulties before exposure of the incident	(1) Yes	89 (24.45)	3.07 ± 0.71	24.68 (<0.01)		2.52 ± 0.84	19.23 (<0.01)		3.14 ± 0.95	4.21 (<0.01)		3.54 ± 0.73	5.16 (<0.01)	
	(2) No	275 (75.55)	2.58 ± 0.83			2.07 ± 0.85			2.64 ± 0.99			3.03 ± 0.99		

Abbreviations: SITMES, Screening scale for Indirect Trauma caused by Media Exposure to Social disaster; M, Mean; SD, Standard Deviation

**Table 2:** The results of final exploratory factor analysis.

No.	Items	Factor 1	Factor 2	Factor 3
3	I used to dream about the incident.	0.77		
5	After the incident, I felt lonely, as if I was alone in the world.	0.77		
7	After the incident, it has become difficult for me to concentrate on everyday tasks, such as reading a newspaper or watching TV.	0.80		
8	I started to avoid news or conversations related to the incident because I do not want to gain more information about it.	0.72		
9	The incident made me suffer even more as I recalled past traumatic experiences.	0.80		
10	After that incident, I have not been able to sleep.	0.91		
11	When I thought of the incident, symptoms such as dizziness, difficulty breathing, cold sweat, and body stiffness occurred.	0.92		
12	After the incident, I lost my appetite.	0.88		
21	I drank to forget my feelings about the incident.	0.80		
23	I tried not to believe the incident happened.	0.55		
24	I worked or performed other activities to forget my feelings about the incident.	0.77		
33	My family members hate when I talk about the incident.	0.75		
35	There was no one with whom I could share my feelings about the incident.	0.70		
38	After the incident, I was disappointed in those who were supposed to support me.	0.80		
39	After the incident, I tried to be alone rather than around other people.	0.64		
16	I was upset when I recalled things or people related to the incident.		0.58	
18	I felt skeptical that the incident took place due to widespread irresponsibility in society.		0.75	
19	Because of the incident, I felt that the world was unjust.		0.92	
20	When I thought about the incident, I felt resentment and indignation.		0.72	
22	1 expressed negative feelings about the incident.		0.48	
1	When I thought about the incident, I felt emotions such as depression, sadness, and fear.			0.67
2	I used to have unwanted painful thoughts or images about the incident.			0.66
13	I thought that something similar to the incident might happen to me or someone close to me.			0.62
14	I became wary of situations, objects, and people similar to the incident.			0.49
Eigenvalues		13.08	2.26	0.96
Explained proportion (%)		52.92	7.85	2.55
Cumulative proportion (%)		52.92	60.77	63.31

Notes: If the factor loading was less than 0.4, it is not shown in this table.

**Table 3:** The results of reliability test.

Factor	No. of Item	Corrected Item-Total Correlation	Cronbach’s Alpha for Deleted Item	Total Cronbach’s Alpha
Factor 1: psychological, physical, and behavioral responses to social disasters	3	0.76	0.96	0.96
	5	0.81	0.96	
	7	0.83	0.96	
	8	0.75	0.96	
	9	0.79	0.96	
	10	0.83	0.96	
	11	0.84	0.96	
	12	0.84	0.96	
	21	0.77	0.96	
	23	0.69	0.96	
	24	0.82	0.96	
	33	0.76	0.96	
	35	0.69	0.96	
	38	0.79	0.96	
	39	0.71	0.96	
Factor 2: moral resentment due to social disasters	16	0.71	0.87	0.89
	18	0.74	0.86	
	19	0.78	0.86	
	20	0.79	0.86	
	22	0.65	0.89	
Factor 3: a sense of threat to life due to social disasters	1	0.71	0.80	0.85
	2	0.72	0.80	
	13	0.67	0.82	
	14	0.67	0.82	
Overall: Screening scale for Indirect Trauma caused by Media Exposure to Social disaster (SITMES)				0.96

**Table 4:** Correlation between SITMES and other measures for criterion validity.

Variables	SITMES	F1	F2	F3
IES-R-K	0.69 *	0.76 *	0.57 *	0.51 *
K-CD-RISC	0.05	0.01	0.05	0.06

Notes: * *p* < 0.01. Abbreviations: IES-R-K, Korean version of the Impact of Event Scale-Revised; K-CD-RISC, Korean version of Connor-Davidson Resilience Scale; SITMES, Screening scale for Indirect Trauma caused by Media Exposure to Social disaster.

## Data Availability

The data presented in this study are available on request from the corresponding author.

## Supplementary Materials

The following are available online at https://www.mdpi.com/1660-4601/18/2/698/s1, Table S1: References for initial items development.
