# Supplementary material for: Development and Evaluation of a Screening Scale for Indirect Trauma Caused by Media Exposure to Social Disasters
Source: Int J Environ Res Public Health. 2021 Jan 15;18(2):698. doi: 10.3390/ijerph18020698 (PMC7830079; doi:10.3390/ijerph18020698)
Supplement: Supplementary file 1 [file ijerph-18-00698-s001.pdf]

International Journal of
Environmental Research and Public Health

MDPI

Table S1. References for preliminary items development.

<table>
<thead>
<tr><th>Concepts</th><th>Components</th><th>References</th></tr>
</thead>
<tbody>
<tr><td rowspan="4">1. Exposure to events</td><td>Media exposure</td><td>• News exposure questionnaire [11]
• Media use questionnaire [16]
• Media exposure questionnaire [24]
• Media Exposure Inventory [31]</td></tr>
<tr><td>Psychological symptoms</td><td rowspan="4">• Items for reasons of psychological impact [13]
• Secondary Traumatic Stress Scale (STSS), Korean version.[30]
• Patient Health Questionnaire-9 (PHQ-9), Korean version [32]
• Primary Care Post-Traumatic Stress Disorder (PC-PTSD) [33]
• Integrated adaptation of Center for Epidemiological Studies Depression Scale (CES-D) [34]
• Brief Symptom Inventory [35]
• Posttrauma Risk Checklist [36]
• Posttraumatic Diagnosis Scale (PDS), Korean version. [37]
• Positive affect and negative affect schedule (PANAS), Korean version [38]</td></tr>
<tr><td>Physical symptoms</td></tr>
<tr><td>Perceived sense of threat to life</td></tr>
<tr><td rowspan="4">2. Individual resilience</td><td>Negative emotional responses</td></tr>
<tr><td>Trauma experience before the event</td><td>• Trauma experience questionnaire [39]</td></tr>
<tr><td>Stress coping method</td><td>• Brief Coping Orientation to Problems Experienced (Brief-COPE) [40]</td></tr>
<tr><td>Meaning in life/ Problem-solving</td><td>• Indirect Trauma Scale of Social Disaster (ITSSD) [23]
• Self-Disclosure Scale [41]</td></tr>
<tr><td rowspan="2">3. Support system</td><td>Family support</td><td>• Family Support Scale [42]</td></tr>
<tr><td>Social support</td><td>• Crisis Support Scale (CSS) [43]
• Duke-UNC Functional Social Support questionnaire (DUFSS), Korean version [44]
• Social Reactions Questionnaire (SRQ), Korean version. [45]</td></tr>
<tr><td>4. Social resilience</td><td>Use of information and services</td><td>• Choi et al. [22]</td></tr>
</tbody>
</table>
